# Development of the organisational health literacy responsiveness (Org-HLR) framework in collaboration with health and social services professionals

**DOI:** 10.1186/s12913-017-2465-z

**Published:** 2017-08-01

**Authors:** Anita Trezona, Sarity Dodson, Richard H Osborne

**Affiliations:** 1 Health Systems Improvement Unit, Centre of Population Health Research, School of Health and Social Development, Deakin University, Melbourne Burwood Campus, 221 Burwood Highway, Melbourne, VIC 3125 Australia; 2 The Fred Hollows Foundation, Melbourne, Australia

**Keywords:** Health literacy, Health literacy responsiveness, Health systems, Health system improvement, Health systems strengthening, Access, Concept mapping, Service improvements, Equity

## Abstract

**Background:**

The health literacy skills required by individuals to interact effectively with health services depends on the complexity of those services, and the demands they place on people. Public health and social service organisations have a responsibility to provide services and information in ways that promote equitable access and engagement, that are responsive to diverse needs and preferences, and support people to participate in decisions regarding their health and wellbeing. The aim of this study was to develop a conceptual framework describing the characteristics of health literacy responsive organisations.

**Methods:**

Concept mapping (CM) workshops with six groups of professionals (total *N* = 42) from across health and social services sectors were undertaken. An online concept mapping consultation with 153 professionals was also conducted. In these CM activities, participants responded to the seeding statement “*Thinking broadly from your experiences of working in the health system, what does an organisation need to have or do in order to enable communities and community members to fully engage with information and services to promote and maintain health and wellbeing”.* The CM data were analysed using multidimensional scaling and hierarchical cluster analyses to derive concept maps and cluster tree diagrams. Clusters from the CM processes were then integrated by identifying themes and subthemes across tree diagrams.

**Results:**

Across the workshops, 373 statements were generated in response to the seeding statement. An additional 1206 statements were generated in the online consultation. 84 clusters were derived within the workshops and 20 from the online consultation. Seven domains of health literacy responsiveness were identified; i) External policy and funding environment; ii) Leadership and culture; iii) Systems, processes and policies; iv) Access to services and programs; v) Community engagement and partnerships; vi) Communication practices and standards; and vii) Workforce. Each domain included 1 to 5 sub-domains (24 sub-domains in total).

**Conclusions:**

Using participatory research processes, a conceptual framework describing the characteristics, values, practices and capabilities of organisational health literacy responsiveness was derived. The framework may guide the planning and monitoring of health service and health system improvements, and has the potential to guide effective public health policy and health system reforms.

**Electronic supplementary material:**

The online version of this article (doi:10.1186/s12913-017-2465-z) contains supplementary material, which is available to authorized users.

## Background

Health literacy refers to the characteristics, skills and abilities required by people in order to access, understand and use information to make decisions about health [1]. Health literacy is an important public health issue, and low health-related reading and numeracy (functional health literacy) has been associated with a range of poor health outcomes [2]. People with low functional health literacy may have less knowledge about their health conditions and treatments, poorer overall health status, and higher rates of hospitalisation than the rest of the population [3–5]. Studies also report an association between low health literacy and a person’s ability to take part in decision-making, to adhere to recommended treatments, to implement health promoting behaviours, and to engage with preventative health services [6–8].

The health literacy skills and abilities required by individuals in order to interact effectively with health services are likely to depend on the complexity of those services, and the demands they place on people [9, 10]. Health systems are complex and health organisations may be structured and operate in ways that make it difficult for people to access and engage with information and health care. For example, services may be located in areas that are difficult to access [11] and may provide unwelcoming and intimidating environments. They may provide information that contains jargon and technical terms, or in ways that do not recognise the language needs, social and cultural background or cognitive abilities of the people they serve [12]. In addition, health practitioners may not be equipped to understand or meet the cultural and social needs of clients, and may not support them to make decisions about their health and wellbeing [13, 14]. These are just some of the health challenges and barriers to health service access that individuals and communities experience.

The interaction between an individual’s health literacy capabilities and the complexity of health systems is now widely acknowledged [10, 15–17]. This has led public health professionals, researchers and policy makers to advocate for the need to address the system level factors that impact on health literacy. That is, health and social care organisations need to improve their health literacy responsiveness. Health literacy responsiveness is a new term in the field of health literacy, and has not been explored empirically. However, it has been described as the ways in which services make health information and support available and accessible to people with different health literacy needs [18]. The concept promotes the responsibility of health care organisations to ensure they meet the health literacy needs and preferences of the people and communities they serve [19–21].

The need to reduce the complexity of health systems and improve the way health care organisations provide information and services was first advocated by the United States Institute of Medicine (IOM) in their 2004 report *Health Literacy: A Prescription to End Confusion* [6]*.* Since that time, efforts to accommodate the health literacy of patients at the system level have largely been advanced through the release of the Health Literacy Universal Precautions (HL-UP) Toolkit, developed by the Agency for Healthcare Research and Quality [22] and a discussion paper by the IOM on what they referred to as a ‘health literate organisation’ [16]. The Universal Precautions approach advocates delivering health care in a way that assumes all clients may have limited health literacy and structuring services in ways that reduce complexity and barriers to access [23]. The HL-UP Toolkit contains a number of tools and resources aimed at addressing health literacy at the organisational level. It focuses on four key areas; verbal communication, written communication, self-management and empowerment, and supportive systems.

In their discussion paper on ‘health literate organisations’, the IOM describes a range of system level factors that health care organisations within the United States health care system should address in order to support people to navigate, understand and use health information and services. The authors proposed a set of ten attributes or goals, accompanied by a set of strategies that organisations can implement. They derived these attributes through expert opinion and a synthesis of the literature on health literacy research and practice. While four of the attributes relate to communication (including specifically on medications, insurance plans and in high risk situations), others include leadership, planning and evaluation, preparing the workforce, involving consumers, and ensuring easy access. The authors stated that they do not see the list of attributes as a definitive response to the challenge of defining a ‘health literate organisation’, rather an optimistic vision of how organisations can be more responsive to the needs of populations. They further stated that the attributes proposed would benefit from further discussion and refinement [16].

Notwithstanding an acknowledgment of the need to further examine and refine the attributes, they are currently being applied within some health contexts outside the US. For example, the ten attributes were incorporated into a key policy statement in Australia [24] as well as informed the development of a health literacy assessment tool in New Zealand [25] and Germany [26]. While the ‘health literate organisation’ concept and attributes have laid important foundations for understanding and describing the system-level factors that need to be addressed in order to respond to health literacy needs, they have not been tested or validated in contexts outside of the United States. Therefore further research on this concept is warranted.

Given individual health literacy capability is dependent on the environment in which the individual is seeking care, it is important that frameworks and recommendations for improving service and system responses are appropriate to the specific social and health care contexts in which they are being applied [18]. Currently there is no framework available that operationalises organisational health literacy responsiveness in the Australian or other contexts, and frameworks that otherwise describe ‘health literate organisations’ are few.

The aim of this study was to empirically develop a conceptual framework on health literacy responsive organisations, grounded in the experiences and perspectives of health and social service professionals. A framework developed in the Australian context, taking into account its complex mix of public health, community health, private health and social structures, is needed to guide policy makers in the implementation of system level reforms and organisational level change. It will also be used to inform the development of organisational self-assessment tools to support organisations with an assessment of their health literacy responsiveness, and subsequently plan, prioritise and evaluate their improvement activities [9, 10, 17].

## Methods

We conducted a series of consultations with professionals working in the health and social services sectors. Firstly, we undertook face-to-face concept-mapping workshops, followed by a two-part online concept mapping process. Concept mapping is an inclusive and participatory research process that can be used to explore community issues. The process actively engages communities as research collaborators, rather than merely as subjects in the research [27] and enables the integration of diverse perspectives and “distributed group knowledge” [28]. It is a mixed methods process that incorporates nominal group techniques, unstructured sorting and multivariate statistical methods [29–31]. The process has been used in public health to develop conceptual frameworks, program logics and questionnaires [32–35]. Concept mapping is useful for establishing content validity, facilitating researcher decision-making and providing insights into participant perspectives [36]. Within the current study, concept mapping enabled a comprehensive and grounded exploration of the potential elements of a health literacy responsive organisation. This study was approved by the Deakin University Human Research Ethics Committee (Study ID: 2012–295).

### Participants and setting

We used purposive sampling to recruit participants to this study, whereby we sought people with experience working across the Australian health and social care systems to address the research aim. To be eligible to participate in a workshop or the online concept mapping process, participants were required to be working in a health (public or private), social or community service organisation in Victoria, or working for a local, state or federal government organisation. We used our extensive professional networks to recruit participants to the workshops. An email invitation was initially sent to approximately 30 people across the health, community, government and research sectors in the state of Victoria, Australia. This included key contacts at Primary Care Partnerships across Melbourne. Primary Care Partnerships are voluntary alliances of health and human service organisations, which work together to improve access to, and coordination of services [37]. These networks further distributed the invitation to their professional networks, which based on the membership of Primary Care Partnerships alone, potentially reached a further 200 organisations and their staff. A total of 42 professionals from 36 organisations participated in six workshops.

Participants in the online concept mapping process were also recruited using a snowball method. An email invitation outlining the online process was distributed to contacts described above, as well as an additional 350 professionals (drawn from the Ophelia Project contact list) across all Australian states and territories. 153 people completed a brainstorming activity and 27 people completed a sorting activity (described below). Table 1 provides a summary of the wide range of organisations/sectors represented in the study, and number of participants for each type (note, participants were able to select more than one type).

**Table 1:** Number of study participants and organisations/sectors represented

Organisation/sector type	Workshops	Online process
Community health/primary care	13	42
General Practice	1	5
Hospital	8	40
Government (Local, State or Federal)	7	24
Non-Government Organisation (NGO)	1	32
Social services	3	13
Health promotion	-	22
Research/education	1	39
Women’s health	1	1
Primary Care Partnership	3	5
Other	4	4

### Materials and procedures

**Fig. 1:**
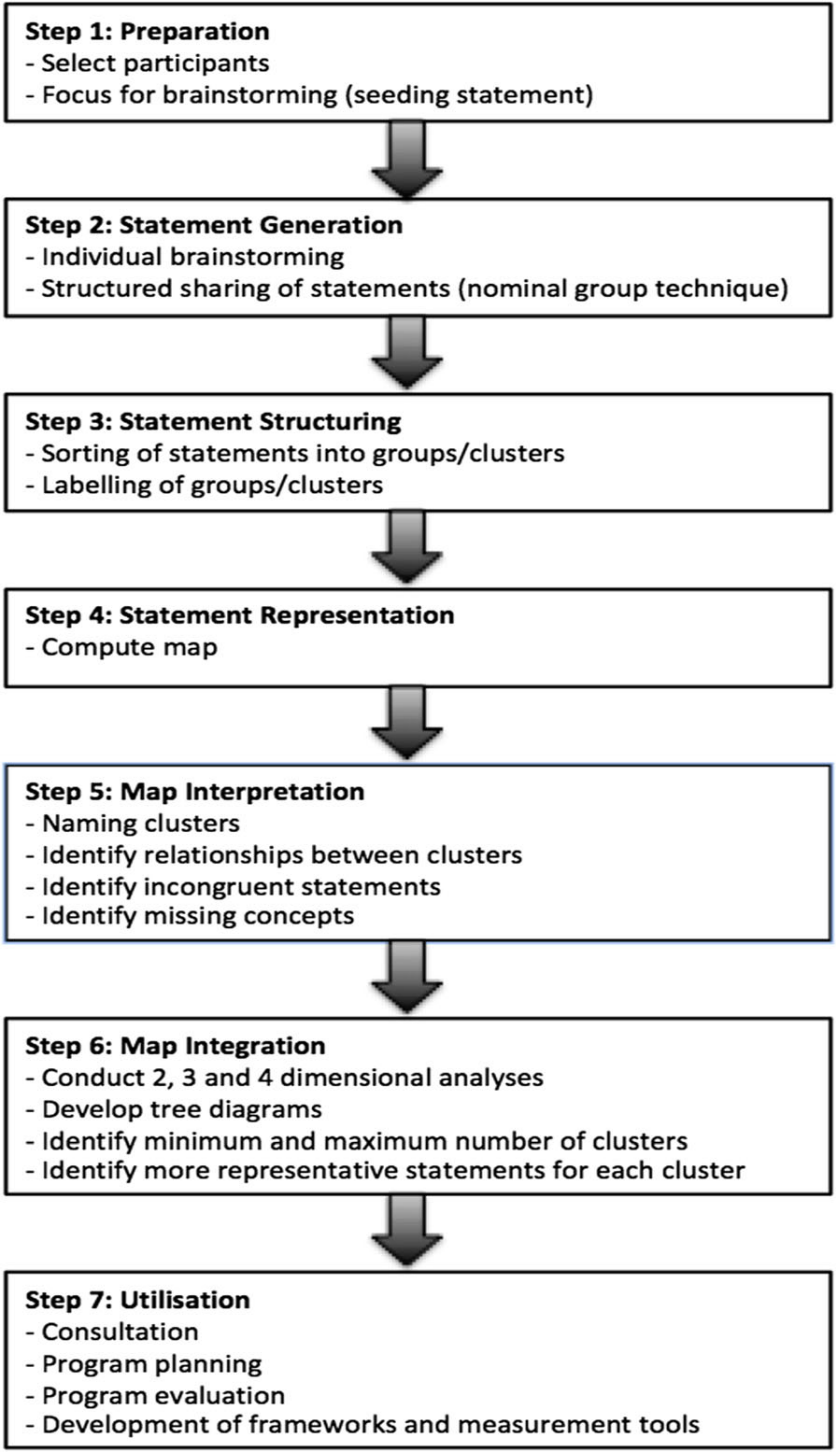
Steps in the Concept Mapping Process. Source: Adapted from van Randeraad-van der Zee et al. 2016

We utilised a modification of Trochim’s approach to concept mapping [29] including the Concept System Software (version 1.0 by Trochim, 1987). This method consists of five workshop steps, followed by two post workshop steps, as shown in Fig. 1. Given health literacy is a multidimensional concept it was necessary to orientate participants to the core dimensions of health literacy, prior to presenting a broad seeding statement relating to engagement with information and services. They were therefore provided with the following information:

*A person’s health literacy is their ability to access, comprehend, appraise, and use information and services to make informed decisions relating to health and wellbeing, and engagement with healthcare and other services *[38]. *The quality of a person’s health-related decisions is influenced by the interaction between their health literacy strengths and weaknesses, and the way the health system responds to people’s needs. Consider health literacy as being influenced by the characteristic of individuals, the service system, and societal and environmental factors.*

We then used the following seeding statement, and summary of the above participant information, in both the workshops and online concept mapping process: “*Thinking broadly from your experiences of working in the health system, what does an organisation need to have or do in order to enable communities and community members to fully engage with information and services to promote and maintain health and wellbeing”.*

#### Concept mapping workshops

To commence each workshop, participants were presented with the seeding statement and asked to work independently to brainstorm as many ideas (statements) as possible in response to it. Participants were then asked to share their statements through a nominal group process led by a facilitator (Step 2). For the structuring step (Step 3), all participants were asked to sort the statements into groups of similar ideas and then assign a label/heading to each group. These headings are used again during the group discussion at Step 5.

In Step 4, a map was computed using the statements and sort data generated by participants into the Concept Systems Software. This is achieved by first entering the statements, verbatim, into the program with a corresponding number. A group number is then recorded against each statement for each participant, according to the way they have grouped the statements at Step 3. The software then combines the groupings of each participant to generate a concept map, using multidimensional scaling (MDS) and hierarchical cluster analysis (using Ward’s algorithm). The MDS positions the statements on a two-dimensional map with x, y coordinates. Statements that appear close together are likely to have been sorted together by participants more frequently. Cluster analysis is then applied to the x, y coordinates to compute cluster boundaries around themes or concepts. These represent the higher order conceptual groupings of the original statements [29]. The position of the clusters on the map is determined by how conceptually similar each of the ideas are, and reveals the strength of relationships between the clusters. It also guides decisions about whether a cluster needs to be split, or whether two clusters should be merged. The software is capable of producing any number of clusters, but for efficiency the default setting was applied (determined as approximately 1/5 of the number of statements) for each workshop.

In the final workshop step (Step 5), the cluster map was presented to participants, along with a list of the statements categorised into clusters corresponding with the map. Through a group consensus process, participants assigned a label to each cluster and discussed possible missing themes. In some instances, participants agreed to move incongruent statements to a more representative cluster.

#### Online concept mapping process

The concept mapping steps outlined above were adapted and incorporated into a two-part online process. Part one of the process was administered using Survey Monkey [39]. The survey incorporated concept mapping Step 2 by asking participants to generate as many statements as possible to the study ‘seeding statement’. The survey also asked participants to indicate whether they were willing to participate in part two of the online process.

Duplicate statements were deleted and the remaining statements underwent minor editing, where necessary, to ensure they were grammatically sound and easily understood by a broad audience. The final list of statements was used in part two of the online process. Each statement was assigned a number and printed onto a separate card. Each participant in part two of the online process was mailed a deck of statement cards along with instructions on how to complete the sorting and labelling tasks (Step 3). They were also provided with a reply-paid envelope to return their sorted and labelled cards to investigators. We used the returned cards to complete steps 4 and 5 of the concept mapping process.

### Data analysis and integration

The data analysis performed by the Concept Systems software focuses on the strength of relationships between ideas. This is a useful and efficient way of generating maps to engage participants in the conceptualisation process during a workshop. However, in order to explore the concepts in greater depth, we undertook further analysis using IBM SPSS Statistics (Version 23.0 by IBM Corporation, 2015). We combined the concept mapping data derived from the workshops and online process into SPSS to perform two, three and four-dimensional scaling (Step 6). We then applied hierarchical cluster analysis (using Ward’s algorithm) to produce a tree diagram comprised of three to 20 cluster solutions. There is no established approach to determining a correct or preferred number of clusters [34], however to determine the key elements of organisational health literacy responsiveness in great detail, we increasingly split the clusters until we reached the maximum number of clusters that made conceptual sense to us.

To integrate the concept map data, rigorous qualitative processes were undertaken. Concept mapping ensures that researcher subjectivity in the interpretation of concepts is minimised, because the statements each contain a singular concept and ambiguous elements are clarified by participants at the point of conception. The groupings across the concept maps are also ‘organised’ by the workshop participants, and similarities between groupings for the workshops and online process were substantial. Our task during the integration process was to preserve distinctions (i.e. minimise concatenation) so that unique ideas were not lost. Through a consensus process the researchers involved in the facilitation of workshops analysed the clusters to identify themes, and then labelled the clusters, as represented by the statements contained within them. Importantly, we continuously cross-referenced these cluster labels with those assigned by participants during the workshops and online process to ensure we retained their ideas and intended meaning. We then developed a mind map to visually display all the derived concepts from each workshop and the online process. Each set of clusters was examined to identify overarching themes, which represented the overarching domains in the mind map. Through an iterative process, we then assigned each cluster to the most appropriate domain, merged conceptually similar clusters, removed duplicate concepts and revised the wording of clusters as appropriate. The remaining clusters represented the sub-domains in the mind map. The mind map was then used to inform the Organisational Health Literacy Responsiveness (Org-HLR) Framework presented in this study.

## Results

### Generation of statements

A total of 373 statements describing the *things an organisation needs to have or do in order to enable people to fully engage with information and services* were generated through the face-to-face workshops. Each workshop generated between 57 and 78 statements. The online concept mapping process generated an additional 1206 statements. After removing duplicate statements, 117 unique statements were identified [the full list of statements are provided in Additional file 1].

### Map generation and interpretation

**Fig. 2:**
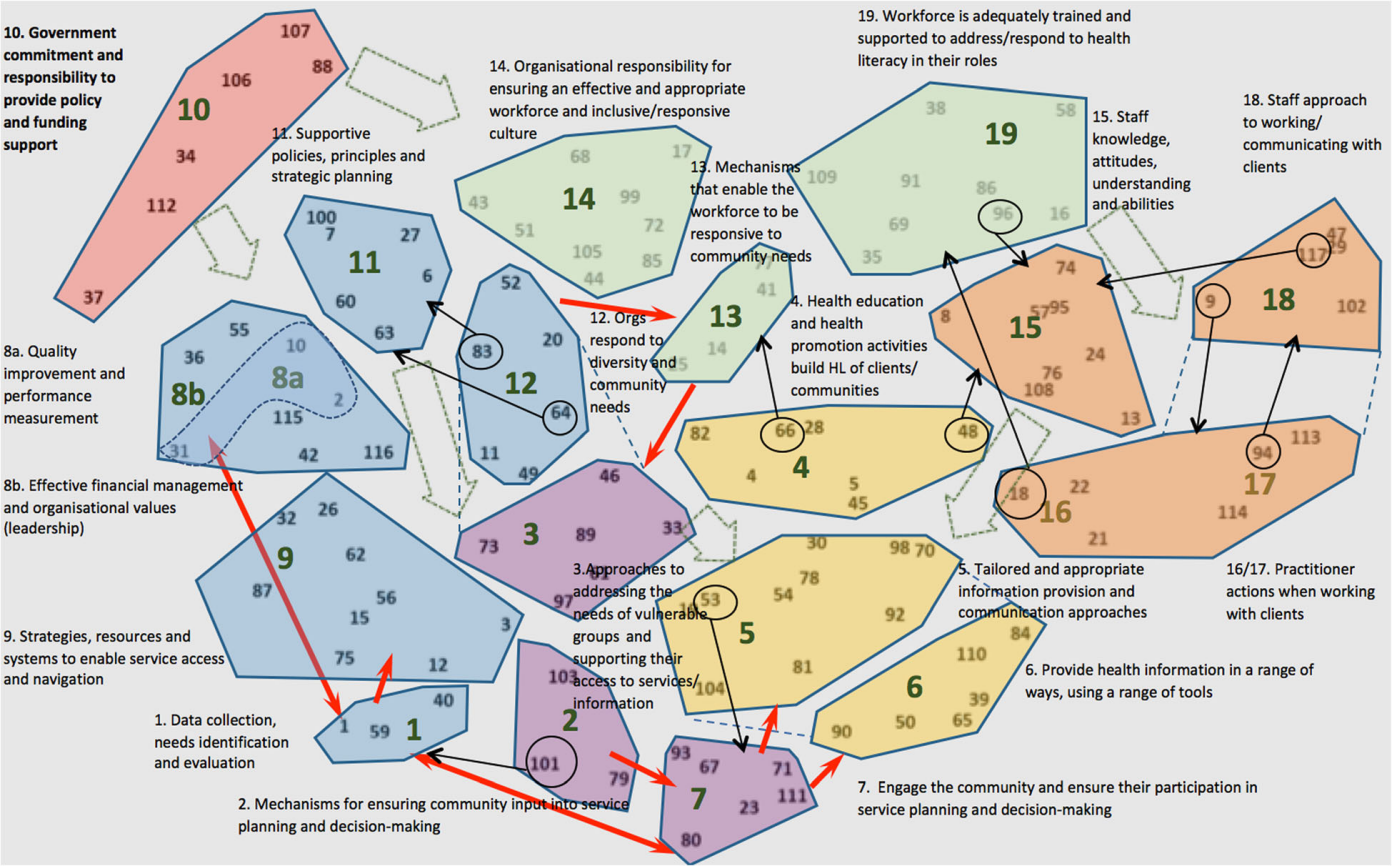
Concept map from online concept mapping process. Legend for interpreting the Map: The large numbers represent cluster numbers, as defined by the cluster borders/shape around it. Each cluster was assigned a label by the investigators based on item content. The circled statements with *black* arrows to another cluster were considered to have content that related to the content of statements in indicated cluster. The *red* arrows show a strong relationship between clusters, and the *green* block arrows illustrate potential influences between clusters. The clusters have been coloured according to an overarching theme, for example the yellow clusters all relate to communication

Figure 2 shows the concept map derived from the online concept mapping process, as an example of the final output of Concept Systems (the colouring has been added to facilitate interpretation of the map). The 20 clusters generated through the online process could be broadly categorised into six overarching themes (Fig. 2). Cluster 10, commitment and responsibility of governments to provide organisations with policy and funding support sits high on the map, with a close relationship to the blue clusters *(clusters 1, 8a, 8b, 9, 11 and 12)*, which collectively represent the themes of organisational leadership, culture, systems and processes. The red cluster and the blue clusters have a link with the green clusters *(clusters 13, 14 and 19)*, which relate to the organisation’s responsibility to ensure a skilled and well-supported workforce. The green clusters in turn have a link with the orange clusters *(clusters 15, 16, 17 and 18)*, which relate to the characteristics, abilities and practices of the workforce, as well as the yellow clusters *(clusters 4, 5 and 6)*, which relate to communication, and the purple clusters *(clusters 2,3, and 7)*, which relate to engaging the community. In a broad sense, the clusters on the top left side of the map can be seen as enablers of the clusters that sit on the bottom right of the map, with the central clusters representing the interface between the workforce and the community/consumer.

This type of positioning of clusters and themes was similar across the six workshop maps. Whilst they did not always sit in the same quadrants, or flow in the same direction, themes and clusters relating to organisational systems, processes and policies tended to be grouped close to clusters relating to leadership and culture as well as to workforce. Themes and clusters relating to workforce in turn tended to be grouped close to clusters relating to communication, which were often grouped close to clusters relating to engaging with the community.

### Map integration

The number of clusters generated for each workshop ranged from 6 to 14 (69 clusters in total). After applying multidimensional scaling and hierarchal cluster analysis, we found the number of clusters that made the most conceptual sense ranged from 9 to 17 clusters across the workshops (84 clusters in total). We then undertook content analysis of these 84 clusters, plus the 20 clusters from the online process to group clusters with like content to form a mind map. For example, clusters such as ‘provide clear and tailored information’, ‘strategies for communicating effectively with the community’, ‘culturally sensitive communication’ and ‘health education caters for varying learning styles’ were grouped together under the broad theme of ‘Communication practices and standards’. Clusters such as ‘health literacy is a strategic priority and organisational value’, ‘the organisation promotes equity’, ‘organisational culture is inclusive and person-centred’ were grouped under the broad theme of ‘Leadership and culture’. We identified seven overarching domains, each with 1 to 5 sub-domains representing organisational health literacy responsiveness. These are shown in Table 2, with an exemplar statement for each sub-domain.

**Table 2:** Domains, sub-domains and exemplar statements of Organisational Health Literacy Responsiveness

Domains	Sub-Domains	Exemplar statements
1. External policy and funding environment	1.1. External policy and funding environment	There are policy frameworks available to guide health literacy work within our organisation
2. Leadership and culture	2.1. Financial management	Health literacy improvement activities are resourced over the long term
	2.2. Leadership and commitment	Managers and decision makers are committed to leading change and health literacy improvements activities across the organisation
	2.3. Health literacy is an organisational priority	Our organisation has clearly defined health literacy goals and objectives
	2.4. Equity and diversity focused	Equity and diversity principles are embedded into organisational plans and policies
	2.5. Consumer-centred philosophy	There is a commitment to delivering consumer-centred care at all levels of our organisation
3. Systems, processes and policies	3.1. Data collection and community needs identification	There is a mechanism in place for determining the health literacy needs of clients and the community
	3.2. Performance monitoring and evaluation	Our organisation’s performance indicators include measures on our health literacy practice
	3.3. Service planning and quality improvement	Our organisation undertakes quality improvement activities/projects for health literacy
	3.4. Communication systems and processes	Handover procedures between practitioners incorporate notes on the health literacy needs of clients
	3.5. Internal policies and procedures	We have policies and procedures in place to support equitable access to services
4. Access to services and programs	4.1. Service environment	Our buildings and venues/facilities are accessible (e.g. affordable parking, ramp access, and close to public transport)
	4.2. Initial entry and ongoing access	We have clear access and referral pathways in place
	4.3. Outreach services	We utilise appropriate support workers to deliver services within the home and community
5. Community engagement and partnerships	5.1. Community consultation and consumer participation	Our organisation consults with the community to develop an understanding of their health and health literacy needs
	5.2. Partnerships with other organisations	Our organisation works collaboratively with service partners to co-design services, programs, materials and referral pathways
6. Communication practices and standards	6.1. Communication principles/standards	We tailor our written and verbal communication to the specific needs of our target groups (e.g. culture, age, gender, sexuality, cognitive abilities etc.)
	6.2. Health information provision	We provide health information using processes that support individual clients learning preferences
	6.3. Use of media and technology	Our website can be accessed in languages commonly spoken in our service region
	6.4. Health education programs	We deliver health education and promotion initiatives that aim to build the health literacy of the community
7. Workforce	7.1. Recruitment	Our organisation has established a set of health literacy competencies required by staff
	7.2. Supportive working environments	Our clinical services are structured in a way that provides practitioners with adequate time to undertake their work effectively
	7.3. Practice tools and resources	Our clinicians are provided with decision-making tools and frameworks to support them with their health literacy practice
	7.4. Ongoing professional development	Our organisation regularly assesses the knowledge, skills and competencies of staff in relation to health literacy

### Description of the domains

Domain 1, External policy and funding environment, relates to the role of governments and other relevant bodies in providing adequate program funding, flexible service agreements, incentives (for example, through accreditation), and health literacy-specific policy frameworks and standards.

The ‘Leadership and culture’ domain (domain 2) describes the necessary ethos, philosophy and values of a health literacy responsive organisation, which includes being inclusive, person-centred and equity driven. It also emphasises the role of organisational leaders and decisions makers to drive effective financial management, service planning, change management and continuous quality improvement.

Domain 3 describes the ‘Systems, processes and policies’ required within an organisation to ensure effective service and program planning, effective internal and external communication, performance monitoring, evaluation and continuous quality improvement.

Domain 4, ‘Access to services and programs’, describes the need for organisations to ensure that services are accessible to all people (physically, geographically, financially and culturally) and emphasises the need to implement strategies that support people to navigate the health system, as well as undertake effective outreach.

Domain 5, ‘Community engagement and partnerships’ describes the need for organisations to undertake meaningful consultation and involve consumers and communities in all aspects of service planning, delivery and evaluation. It also emphasises the importance of engaging and developing partnerships with a range of health, social service and specialist organisations to improve service coordination and strengthen program planning and delivery.

Domain 6, ‘Communication practices and standards’ describes the broad range of strategies and approaches for effective communication across all levels of the organisation, including health education activities and the effective use of new media and technology. It also describes principles and standards for ensuring that written and oral communication is accessible, inclusive, respectful, and tailored to the specific health information needs and learning styles of clients and communities.

Finally, the ‘Workforce’ domain (domain 7) describes the responsibility of organisations to ensure they maintain a competent workforce by recruiting staff with the appropriate skills, knowledge and attitudes, as well as providing a supportive working environment, practice resources and professional development opportunities.

## Discussion

This is the first empirical study to conceptualise health literacy responsiveness, developed through an inclusive and collaborative process involving professionals working in the health and social services sectors. Nearly 200 stakeholders contributed to the development of the distinct elements we describe as the Organisational Health Literacy Responsiveness (Org-HLR) Framework.

**Fig. 3:**
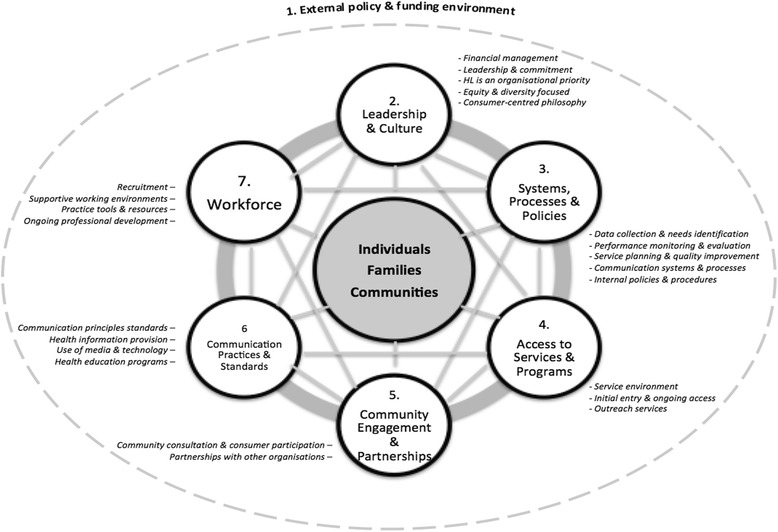
The Organisational Health Literacy Responsiveness (Org-HLR) Framework

The Org-HLR Framework comprises seven domains and 24 sub-domains, as depicted in Fig. 3. The dashed outer line in the diagram represents the organisation or the health system. It was necessary to include this to show that domain 1, the *External policy and funding environment* sits outside of the organisation and is not within the organisation’s direct control*.* This domain acknowledges that a supportive policy and funding environment influences organisational capability and functioning, and is a key enabler of organisational responsiveness.

The six domains sitting inside the dashed outer line describe the characteristics values, practices, and capabilities that organisations require in order to effectively respond to the health literacy needs of their service users and broader communities. These domains are presented as connected circles to highlight the interconnected nature of these elements. The *Leadership and culture* (domain 2) within an organisation influence the extent to which *Access to services and programs* (domain 4) is supported, as well as the *Systems, processes and policies* (domain 3). Together, these may be seen as enablers of the *Workforce* (domain 7), and the foundations for effective *communication* (domain 6) and *engagement* with individuals, communities and other stakeholders (domain 5).

The figure also seeks to highlight the interconnection between the domains, i.e., the relationships and processes are not linear. It recognises, for example, that the workforce is an important driver of organisational leadership and culture, and is also instrumental in ensuring the effective implementation of systems, processes and policies. Similarly, it describes that when communication and engagement are undertaken effectively, organisations ensure that service users, communities and stakeholders have the opportunity to inform all aspects of the organisation, including its culture, its systems, processes and policies, and the effectiveness of its workforce. Public health and social service organisations exist to serve the needs of individuals, families and communities, therefore organisations must engage them and consider them in all aspects of service and program design, delivery and evaluation. Hence, the domains of health literacy responsiveness are connected around a central node representing ‘individuals, families, communities’, to highlight their centrality in the conceptualisation of health literacy responsiveness.

The intention of this study was to conceptualise health literacy responsiveness and provide a solid foundation on which this concept can be taken forward in future research, service improvement, promotion of equity and access, and policy making. We argue that health literacy responsiveness is a meaningful way of describing the aim of system level efforts to address health literacy as it emphasises an orientation towards action and the need for organisations to be proactive in meeting community needs. Given the empirical findings from extensive consultation across the sectors and health care providers described in this paper, we define health literacy responsiveness as:

*The provision of services, programs and information in ways that promote equitable access and engagement, that meet the diverse health literacy needs and preferences of all people, and that support individuals and communities to participate in decisions regarding their health and wellbeing, which is achieved through supportive culture and leadership, supportive systems, policies and practices, and an effective workforce.*

In-depth consultation has revealed that health literacy responsiveness is achieved through an organisational culture that promotes equity and inclusiveness; effective organisational leadership and management; robust systems, processes and policies; a strong commitment to engaging with communities and collaborating with other organisations; developing and supporting an appropriate and capable workforce; and through effective communication principles and practices.

This study builds on the foundations laid by the IOM’s concept of a ‘health literate organisation’. In their discussion paper, the authors encourage further exploration and refinement of the ‘ten attributes’. This study has provided confirmation of a number of their proposed attributes. For example, the *Leadership and culture* domain of the Org-HLR is expressed as ‘leadership promotes’ in the IOM attributes. The *Access to services and programs* domain includes similar concepts to the ‘ensures easy access’ component. The IOM include a dimension on involving consumers and meeting the needs of all populations, which aligns broadly with the *community engagement and partnerships* domain of the Org-HLR. Finally, the Org-HLR Framework shares some overlap with the workforce and communication dimensions of the IOM’s attributes. The Org-HLR provides fine-grained operationalisation of the general components of health literacy responsiveness and the IOM attributes.

Given the wide engagement with stakeholders undertaken to generate the Org-HLR Framework, the new dimensions identified suggest new areas to be considered in endeavours to address system level factors that impact on health literacy. The first is the *External policy and funding environment* domain. This is an important element as it recognises that policy and funding can either enable or constrain organisations in their endeavours to be more responsive to the health literacy needs of their clients and communities. During our consultations with professionals, participants frequently stated that organisations look to government policies to guide their planning and priority setting. They acknowledged that organisations are only willing to deliver the programs and services that are stipulated in service agreements, and only implement service improvements that are outlined in accreditation standards. Therefore, in order to be responsive to health literacy needs, organisations require policies that guide effective planning and priority setting, and funding agreements that allow for flexible service and program delivery.

The *Systems, processes and policies* domain of the Org-HLR expands on the IOM planning and evaluation attribute to provide a comprehensive set of system capabilities and internal policies an organisation needs to have in place. The Org-HLR also provides an expanded operationalisation of the workforce dimension, outlining four key sub-components that make up and effective and well-supported workforce; recruitment, supportive environments, practice resources and ongoing professional development.

Finally, an important new dimension described in the Org-HLR is the component on partnerships with other organisations. The partnerships component of the Org-HLR is critical, given the complexity of the systems health care organisations are required to operate within. Partnerships and collaboration are essential for enabling health care organisations to effectively coordinate and integrate care for individuals, and adequately support them on their journey through the health system [40, 41].

The Org-HLR describes a comprehensive range of characteristics values, practices, and capabilities that make up a health literacy responsive organisation. Its domains and sub-domains provide a coherent structure for approaching the organisational improvements necessary to embody the concept of health literacy responsiveness. A key strength of this framework is the participatory method used in its development. The concept mapping approach allowed us to integrate input from a diverse range of professionals working in the health and social services sectors. By engaging these professionals as collaborators in the conceptualisation of health literacy responsiveness, the Org-HLR is more likely to reflect the operational environments across these sectors and therefore more likely to be taken up and utilised in the planning and implementation of programs, services and improvement activities in the future [42, 43].

This study had two main limitations. Firstly, while participants were central to the conceptualisation of health literacy responsiveness, by generating the statements, cluster groupings and cluster names during the concept mapping process, the end-stage conceptualisation and final framework development was led by the research team, and we did not undertake a consultation with these stakeholders to confirm the final domains and sub-domains of the framework. However, the framework will be used to operationalise a self-assessment tool, which will be tested with health and social care organisations in several countries. This will provide information on how the Org-HLR may need to be tailored to local health settings.

Secondly, as this study forms part of a broader Victorian study on health literacy and system improvements [44], it was necessary to consult extensively with professionals working in Victoria. While we did also engage participants from across other parts of Australia, the large number of Victorian participants may have resulted in the generation of concepts that are more specific to health systems similar to the Victorian than to other health systems. That is, health systems that are comprised of a mix of publicly and privately funded services, with large tertiary organisations, public and private primary care providers and a strong community health sector.

The Org-HLR Framework has a wide range of potential applications. Policy makers may utilise it in the development and monitoring of policies relating to health literacy and health system reform, as well as the quality and safety accreditation standards that govern health services. Health and social service organisations may utilise it to inform quality improvement and organisational development activities. It has the potential to inform the development of training and education curricula for health and social service sector professionals, and researchers may utilise it to inform research programs on health literacy responsiveness and health system strengthening. The Org-HLR Framework has already informed the development of an organisational self-assessment tool and planning resources to support organisations assess their health literacy responsiveness and plan their health literacy related improvement activities.

## Conclusions

We utilised a participatory research process to develop a conceptual framework that describes the characteristics, values, practices, and capabilities of a health literacy responsive organisation, in collaboration with health and social service sector professionals. The framework provides a coherent structure for identifying, planning and monitoring health service and health system improvements, and has the potential to guide effective public health policy and reforms to enhance the health literacy responsiveness of health services and systems.

## Additional file

Additional file 1: Online concept mapping statements. This file contains the list of 117 unique statements that were identified by the research team, after removing duplicate statements from the 1206 statements generated by participants during the online concept mapping process. (XLSX 14 kb)

## Abbreviations

CM: Concept mapping
HL-UP: Health Literacy Universal Precautions
IOM: Institute of medicine
MDS: Multidimensional scaling
Org-HLR: Organisational Health Literacy Responsiveness
PCHS: People-centred health services
WHO: World Health Organization
